# Microarray patches as a potential alternative to injectable fertility medications: a qualitative study of patients’ and healthcare professionals’ perceptions

**DOI:** 10.1007/s11096-026-02220-1

**Published:** 2026-09-23

**Authors:** Laura Koenitz, Megan Quigley, Abina Crean, Sonja Vucen, Michelle O’Driscoll

**Affiliations:** 1 SSPC, the Research Ireland Centre for Pharmaceuticals, School of Pharmacy, University College Cork, Cork, Ireland; 2 School of Pharmacy, University College Cork, Cork, Ireland; 3 Pharmaceutical Care Research Group, School of Pharmacy, University College Cork, Cork, Ireland

**Keywords:** Fertility, Healthcare professionals, Injections, Microneedle, Patients

## Abstract

**Introduction:**

Many fertility treatments require regular injections that can impact patients’ everyday lives. Transdermal delivery may reduce the treatment burden, but physical and biochemical barriers of the skin limit the drugs’ bioavailability. Microarray patches (MAPs) present a novel formulation option that helps delivering medicines through the skin. Their administration is perceived to be straightforward and patient-friendly; however, existing acceptability studies have largely focused on MAP-based vaccination.

**Aim:**

This study aimed to investigate patients’, partners’ and healthcare professionals’ experiences of fertility injections, and to explore their perspectives on potential MAP use and key factors for optimal user acceptance in fertility care.

**Method:**

A qualitative interview study was conducted in Ireland between September 2025 and January 2026. Patients, partners and healthcare professionals (including nurses, pharmacists, midwives and consultants) were eligible to participate. Interviewees were recruited by convenience sampling through researchers’ networks, advertisement on social media and targeted outreach to fertility clinics. Semi-structured interviews followed a topic guide. Audio recordings were transcribed verbatim and transcripts analysed using Braun and Clarke’s reflexive thematic analysis.

**Results:**

Twenty-two participants (n=10 patients/n=1 partner, n=11 healthcare professionals) of varying age, professional/educational background, residential/practise location, and experience with fertility care were interviewed. Five key themes emerged: (1) The complexity of administration-drug and formulation considerations; (2) The importance of support systems-partners, peers, and healthcare professionals; (3) Managing expectations-medicine information and treatment outcome; (4) Making it work-logistical and lifestyle adjustments; and (5) Education and training-healthcare professionals and patients. Participants highlighted patients’ hopes for treatment success and their resilience to endure practical and emotional challenges like reconstituting injectables, sharps disposal, and fear of mistakes. While MAPs could enhance treatment experiences, they should meet important acceptability criteria including straightforward use, flexible dosing, room temperature storage, minimal pain and skin reactions, comparable efficiency and cost to injectables, and comprehensive counselling.

**Conclusion:**

This study highlighted the potential positive impact of MAPs on the unique practical and emotional challenges of fertility patients. The findings will help to inform next steps in exploring MAP-based fertility treatments but also have implications for the development of future fertility injectables.

**Supplementary Information:**

The online version contains supplementary material available at https://doi.org/10.1007/s11096-026-02220-1.

## Impact statements

- This study identified practical and emotional challenges that patients face when administering, handling and storing fertility injections.
- The potential use of microarray patches may enhance patients’ experience compared to traditional injectables if they fulfill important handling, storage, regulatory, and administrative criteria.
- Comprehensive patient training and strong support systems were identified as key facilitators to a successful treatment.
- This research provides direction for the development of future microarray patch and injectable medicines in fertility care.

## Introduction

Infertility affects approximately 10% of reproductive-aged couples worldwide. [1] In high-income countries up to 20% of them avail of assisted reproductive technologies like in vitro fertilization (IVF) to conceive. [2] In the last decades over ten million infants were born with their help [3] but fertility treatments often place emotional and physical strain on patients. [4, 5] Many protocols require daily injections to induce multi-follicular development by controlled ovarian stimulation or support embryo implantation post-transfer.

Fertility injections may cause pain and physical discomfort. [6] Furthermore, they can impact everyday lives as patients and partners may need to rearrange work schedules or organize their social life around them. [7–9] Patients also worry about making mistakes like giving the wrong dose or preparing the medication incorrectly. [6, 8–10] Some injections require cold storage, and all generate biohazardous sharps waste which can pose logistical challenges for patients. [8].

However, the treatment burden could be reduced by oral or transdermal delivery, less frequent injections, or pre-mixed products. [8, 10–12] While there has been success in making more injections available as prefilled pens [13], the development of oral or transdermal formulations remains a pharmaceutical challenge. Physical and biochemical barriers of the skin and enzymatic degradation upon ingestion result in low bioavailability via these routes due to the drugs’ size and structure. [14].

Microarray patches (MAPs) are a novel formulation option that contain tiny projections called microneedles to puncture the outermost layer of the skin. They are able to deliver drugs through the skin that were traditionally not suitable for the transdermal route. [15] The administration of MAPs is perceived to be straightforward and patient-friendly [16] and only causes minimal discomfort and pain. [17, 18] Depending on the specific formulation, MAPs may increase drug stability at room temperature and thereby simplify storage compared to injections. [19] Furthermore, some MAPs dissolve in the skin during administration and therefore do not generate biohazardous waste. [20].

The acceptability of therapeutic MAPs has been explored through interviews studies, focus groups, surveys, and clinical trials related to vaccines [21–25], therapeutic drug monitoring [26, 27], and drug delivery for contraception and HIV prevention [28–31]. Overall, patients and healthcare providers perceived MAP administration as positive, highlighting ease of use, minimal training requirements, discretion, and advantages for patients with needle phobia. [16, 17, 23, 27, 29–31] Besides, concerns around their efficacy, inability to confirm successful drug delivery, and skin irritation were raised. [16, 27, 29] However, perceptions of MAPs may vary across different therapeutic areas and currently remain underexplored for fertility treatments. Patients and healthcare professionals (HCPs) may have unique needs and perspectives regarding MAP use considering complex treatment regimens and patients’ emotional investment in fertility care, which may differ from findings in prior acceptability studies focused on other indications.

### Aim

This study aimed to investigate patients’, partners’ and HCP’s experiences of fertility injections, and to explore their perspectives on potential MAP use and key factors for optimal user acceptance in fertility care.

## Method

Consolidated criteria for reporting qualitative research (COREQ) [32] were applied as a structured guideline to ensure complete and transparent reporting.

### Participant selection and recruitment

The study sought to include the perspectives of the two key groups involved in fertility care, namely patients/their partners and HCPs including nurses, midwives, and consultants from fertility clinics and community pharmacists. Participants were recruited in Ireland by convenience sampling through researchers’ networks, advertisement on social media and targeted outreach to fertility clinics. To ensure a representative sample was obtained, efforts were made to recruit patients/partners of varying educational background, residential area, socio-economic status, and experienced fertility treatment, and HCPs of different age, gender, practice location, profession and years of experience. To reach a diverse audience, study information was shared on social media and various fertility clinics contacted. Demographics of participants were monitored throughout recruitment to ensure representation of participants with different demographics and experience with fertility care.

### Data collection

All participants took part in a semi-structured interview in person or online using Microsoft Teams. Prior to participation, participants were asked to provide written informed consent and demographic information. The interview comprised of an introduction by the researcher and two main parts described in the topic guides (Supplemental material). In part one, participants were asked about previous experiences of fertility injections. In part two, interviewees received a brief introduction to MAPs including general information on their potential size, administration pain and storage requirements as outlined in topic guides (Supplementary material) and were given the opportunity to ask follow-up questions. Participants were also shown a cosmetic or placebo MAP. This was followed by questions about the participants’ perspectives on potential MAP use in fertility care, including their views on essential properties for optimal patient acceptance. All interviews were audio-recorded and transcribed verbatim. Transcripts were checked independently by two researchers for accuracy prior to deletion of the recordings. Initial notes were taken by the researcher after the interviews.

A practice interview applying the topic guide was conducted with an academic pharmacist prior to the first interview. This interview was not included in the study. Furthermore, the first interview with a participant from each group was carefully reviewed by the research team to identify potential unclear, confusing, or distressing questions. After this review was completed, interviews were included in the study, and the study continued as planned.

Eleven interviews per group were conducted. While an adequate sample size depends on the depth and relevance of gathered data, empirical research studies suggested that high degrees of thematic saturation were generally achieved in 9–17 interviews particularly for relatively homogenous study populations. [33] In this study, nine interviews per group that met the sampling matrix criteria including representation in each cohort were considered to attain sample adequacy. These were followed by an additional two interviews per group to ensure a stable and complete thematic structure and confirm the reliability of the findings.

### Data analysis

This study adopted a qualitative descriptive methodology with an interpretivist perspective. Interview transcripts were analysed using Braun and Clarke’s reflexive thematic analysis. [34] After familiarisation with the data by reading and re-reading all transcripts, initial codes were generated using NVivo software [35]. Coding of all transcripts was conducted by one researcher, while a second researcher independently coded 2/11 (18%) per group to ensure alignment. Once all interviews were coded, generated categories were gradually grouped into subthemes and finally condensed to five key themes. These themes were reviewed and agreed on by all researchers, defined and named. In the final step, findings were prepared for publication relating identified themes to the research questions and existing literature.

### Reflexivity

The research team comprised of three female academics with a background in clinical pharmacy or pharmaceutics, one female PhD researcher practicing parttime as a community pharmacist and a female final-year pharmacy student. The PhD researcher conducted all interviews, transcripts were checked by the PhD researcher and the pharmacy student, and transcripts were coded by the PhD researcher and a clinical pharmacy academic. The PhD researcher received continuous training and support from the clinical pharmacy academic particularly on the conduction of interviews, coding of transcripts including use of NVivo and data interpretation. Researchers recognize that their own experiences of working in community pharmacy and prior professional interactions with patients and healthcare professionals may have influenced how data were gathered during interviews, analysed and interpreted. Throughout the study researchers reflected on and discussed their interactions with participants, choice of methodology and data interpretation. Thereby, their backgrounds in different research areas (clinical pharmacy, pharmaceutics) helped to reduce individual biases and assumptions.

### Ethics approval

This study was approved by the Social Research Ethics Committee of University College Cork (reference number: 2025-109, date of approval: 27th June 2025).

## Results

A total of twenty-two interviews were conducted, with ten patients, one partner and eleven HCPs participating. Interviews lasted between 15–42 min (mean: 24 min) for patient/partner group and 6–27 min (mean: 16 min) for HCP group. In the patient/partner group, most participants lived in a suburban (55%) or rural/semi-rural (36%) area. Only one interviewee had a medical card (9%) which provides free medical services and prescription medicines to Irish residents after a means-tested financial assessment. Furthermore, all interviewees had a university degree, with most completing a Master’s (55%) or PhD (36%) programme. The number of experienced IVF cycles varied from one to five. Full patient/partner demographics are presented in Table 1.

**Table 1:** Patient/partner demographics

Participants’ characteristics		Number of participants interviewed
Patients/partners	Patient	10 [91%]
	Partner	1 [9%]
Gender	Female	10 [91%]
	Male	1 [9%]
Residential area	Urban suburb	6 [55%]
	Rural/Semi-rural	4 [36%]
	City centre	1 [9%]
Highest educational degree	Master	6 [55%]
	PhD	4 [36%]
	Bachelor	1 [9%]
Medical card holder	No	10 [91%]
	Yes	1 [9%]
Number of IVF cycles	1	3 [27%]
	2–3	5 [45%]
	4–5	3 [27%]
Time since last IVF cycle (Years)	< 0.5	5 [45%]
	0.5–2	1 [9%]
	> 2	5 [45%]

The HCP group comprised of four nurses and pharmacists each, two fertility midwives and one consultant. Most participants (91%) were female. Interviewees’ age, years of professional experience, and time working with fertility patients varied, with some joining fertility care in the last four years (27%) and others having more than 20 years of experience (18%). All participants practiced in a city centre (36%) or suburban (64%) location. Full HCP demographics are presented in Table 2.

**Table 2:** Healthcare professional demographics

Participants’ characteristics		Number of participants interviewed
Profession	Nurse	4 [36%]
	Pharmacist	4 [36%]
	Fertility midwife	2 [18%]
	Consultant	1 [9%]
Gender	Female	10 [91%]
	Prefer not to say	1 [9%]
Practice location	Urban suburb	7 [64%]
	City centre	4 [36%]
Age (Years)	18–29	1 [9%]
	30–39	5 [45%]
	40–49	2 [18%]
	≥50	3 [27%]
Professional experience (Years)	0–4	1 [9%]
	5–9	3 [27%]
	10–19	4 [36%]
	≥20	3 [27%]
IVF experience (Years)	0–4	3 [27%]
	5–9	2 [18%]
	10–19	4 [36%]
	≥20	2 [18%]

Five key themes emerged during analysis comprising relevant practical and emotional aspects of injectable and potential MAP use in fertility care as presented in Table 3. Selected participant quotes are embedded in the following sections with additional quotes provided in Table S1 (Supplemental material).

**Table 3:** Patients’ and HCP’s perspectives on injectable and MAP use in fertility care: Themes, their definition and key aspects

Theme 1: The complexity of administration - drug and formulation considerations Definition: Characteristics of pharmaceutical products and their administration by patients		
*Practical aspects:*	*Emotional aspects: *	*Potential MAP use:*
Injection type (Intramuscular, subcutaneous)	Control over medication preparation/administration	Easy administration
Medication preparation (Reconstitution)	Needle phobia	Pain/needle-free option
Injection site reactions	Fear of mistakes/product errors	Less human/technical errors
	Independence (Self-administration)	Safety of MAP excipients
		Administration site reactions
Theme 2: The importance of support systems - partners, peers, and healthcare professionals		Flexible dosing
Definition: Care from patients’ social and medical environment.		
*Practical aspects:*	*Emotional aspects: *	*Potential MAP use:*
Help with injections (i.e. from partner)	Support person during first injections	Less dependent on help from others
Advice and encouragement from HCP	Relationship impact (Tension, opportunity for partner to get involved)	Partner involvement
Practical tips from peers		
Theme 3: Managing expectations - medicine information and treatment outcomes Definition: Patients’ anticipation of medication and intervention results.		
*Practical aspects:*	*Emotional aspects: *	*Potential MAP use:*
Medicine labelling/unlicensed use	Hope for treatment success/self-blame for failures	Hesitation towards new medicines
Medicine information	Resilience/commitment	Efficiency concerns/MAP adhesion
	Aversion to treatment changes	Label without ‘needle’
Theme 4: Making it work - logistical and lifestyle adjustments Definition: Incorporation of medicines in patients’ daily schedule.		
*Practical aspects:*	*Emotional aspects: *	*Potential MAP use:*
Injection routine	Privacy/discretion	Refrigeration/sharp removal (Logistics/stress↓, privacy↑)
Injection in public	Exhaustion from logistical requirements	Reimbursement of MAPs
Cold storage		Waste reduction
Sharps disposal		
Treatment cost		
Environmental impact		
Theme 5: Education and training - healthcare professionals and patients Definition: Knowledge transfer to healthcare professionals and patients.		
*Practical aspects:*	*Emotional aspects: *	*Potential MAP use:*
Patient counselling (Demo sessions, online resources)	Counselling to reduce anxiety	Easier and quicker training compared to injections
HCP education/knowledge	Patient distrust in HCPs due to poor advice	Counselling on potential treatment errors
Interprofessional cooperation between HCPs		

### Theme 1: The complexity of administration - drug and formulation considerations

HCPs reported that patients generally handled fertility injections well, especially after an adjustment period. Actively taking part in preparing and administering injections gave patients comfort as it was something they could control. However, complex treatment regimens that involved multiple types of medicines posed challenges. Additionally, injection site reactions like pain, bruises, and lumps from oil-based preparations were common concerns among patient participants. Most patients did not mind subcutaneous injections with prefilled pens but struggled with gluteal intramuscular administrations due to their positioning requirements and large needles.

‘*Physically trying to reach around to the right area [for gluteal injections]… trying to kind of inject into a mirror involved in a certain level of dexterity that I didn’t have.*’ (PAT9, patient).

HCPs and patients were generally interested in MAPs as an alternative to injections and positively highlighted their potential painless, needle-free and straightforward administration. Some patients and HCPs questioned MAP’s benefits over short-term subcutaneous but saw their relevance compared to long-term intramuscular injections. Concerning MAP characteristics, patients were open to long wear times, different administration sites and patch sizes if MAPs were covered and no pain was experienced. Some patients described a comforting feeling after a successful injection and suggested a MAP delivery indicator for a similar experience. HCPs and patients were interested in MAP excipients and their safety profile. Some worried about MAP residues remaining in the body. HCPs emphasized the importance of flexible dosing and advantages of sustained-release MAPs.

‘*[T]o put a patch on every two days, twice a week, once a week… would be a dramatic improvement.*’ (HCP11, pharmacist).

Furthermore, many patients discussed feeling anxious about making mistakes during the preparation and administration of injections. Few reported stressful experiences with product failures like stalling pens or leaking syringes. Besides being afraid of causing harm, patients worried about impacting the outcome of their cycle, a fear that was further accelerated by patients’ financial investment in fertility treatments. Some patients anticipated less human and technical errors with MAPs due to their straightforward use.

‘*The mindset of the person taking it… they are kind of vulnerable and they are already feeling like their body has let them down… a mistake or an error in that context can just feel really amplified.*’ (PAT4, patient).

### Theme 2: The importance of support systems - partners, peers, and healthcare professionals

Devices like prefilled pens are designed for self-administration, so HCPs recommended patients to self-inject. While most patients did so independently, some got help from partners, family members, and friends, especially with challenging intramuscular injections. However, passing injections on to another person may add logistical complexity as highlighted by HCPs. Participants imagined that MAPs could reduce patients’ dependency on others for help and enhance their confidence in self-administration.

‘*If they’re going to involve a partner in doing things, it becomes even more complicated of course, because you are trying to get two people in one place at the same time every day… that's not easy to organise in busy dual income lives.*’ (HCP11, pharmacist).

Furthermore, the impact of fertility injections on couples’ relationships was complex. While helping with the injections was an opportunity for partners to get involved, it may cause tension as highlighted by HCPs. Also, injections medicalized the natural process of conceiving for one partner. Therefore, an interviewed nurse recommended partners to support in other ways like ‘*booking nice things… adding the softness… making the house comfy’* (HCP5). Participants imagined that MAPs may promote self-administration; however, one partner highlighted that they could still get involved in MAP applications.

‘*I would administer it to her… But the times where I couldn't and she had to do that to herself like they were difficult times. So having something that's a bit less yeah needily would be good.*’ (PAT1, patient partner).

Furthermore, HCPs and patients emphasized the emotional value of a strong support system like having another person present during the first injections. Moreover, the advice and encouragement from HCPs was critical to many patients with some highlighting the value of out-of-hours support for urgent queries. Practical tips from peers through online forums were another important resource to patients.

### Theme 3: Managing expectations - medicine information and treatment outcome

Interviewees highlighted patients’ hopes for a positive outcome, and the strength, resilience and commitment they drew from it. Multiple patients reported that they were willing to ‘*do whatever it takes in whatever form it comes in’* (PAT10). For many, the outcome of fertility treatments was most important, while convenience of administration was secondary. High expectations posed pressure on some patients and made them question their own accountability for unsuccessful cycles. One pharmacist noted that fertility patients tended to *‘attribut[e] blame very quickly as opposed to understanding what's going on’* (HCP11).

‘*But at the end of it all, you're doing that process in order to get the best efficiency… If you have to stick the needles into yourself, you do it in order to get it.’* (PAT9, patient).

Hence, efficiency was the key criterium for MAP acceptance among patients. Multiple HCPs and patients expressed concerns over low absorption from MAPs particularly in people with a high body mass index. Others would not question MAP efficacy if licensed. Participants from both groups discussed adhesion issues that could raise efficiency questions. Moreover, interviewees noted that an adjustment period was required for MAPs as fertility treatments were associated with injections, clinics relied on established protocols and patients were apprehensive to new medicines. Nevertheless, some HCPs emphasized patients’ trust in their recommendations and the familiarity with medicated patches in fertility care. One patient noted that irregularities may be blamed on a new medicine like MAP, and that success stories from other patients were more relevant to them than scientific information.

*‘I wouldn't have liked if something felt kind of experimental or we're trying out these new patches… you'd kind of just want everything to work.’* (PAT10, patient).

Additionally, clear medicine information and labelling were crucial. The label ‘Use in men only’ on a buserelin injection due to its license for prostate cancer caused a patient ‘*a little bit of anxiety and maybe a bit of confusion’* (PAT5). Hence, patients highlighted that potential MAP products should be licensed for fertility treatments and MAP labels should avoid the word needle as it may confuse needle-phobic patients. Furthermore, some patients discussed their hesitation to change treatment protocols, for example during medicine shortages. Involving them in treatment decisions and acknowledging their emotions was an integral part of managing expectations.

‘*I suppose when I felt like I had an input to an extent in what I would be taking, I felt a little bit more ownership over the process.*’ (PAT5, patient).

### Theme 4: Making it work - logistical and lifestyle adjustments

HCPs highlighted that many patients had an aversion to needles when starting treatment but gained confidence in injecting and developed a routine quickly. Most patients preferred injecting at home. Others had no issues bringing injections to public spaces but highlighted the need for planning. Both HCPs and patients emphasized the importance of staying independent through self-administration, especially for long-term injections. For many patients, logistical requirements added to emotional and physical exhaustion from fertility treatments.

‘*You have the financial side, the emotional side, the physical side, trying to get to all appointments, remembering all your medication… It is just really a draining process.*’ (PAT5, patient).

Most interviewees agreed that cold storage and sharps handling was inconvenient at times but generally not difficult to manage. Some patients struggled with the amount of space that injections occupied in their fridge and HCPs reported regular queries on medicine refrigeration during travels. However, patients handled it well after counselling. Furthermore, a pharmacist highlighted that short-term room temperature storage of some refrigerated injectables was a major facilitator ‘*for people who are travelling… are on shift changes… want to go away for the weekend*’ (HCP11).

Regarding waste, some patients struggled to find a pharmacy that accepted their sharps bin or were frustrated over charges for its disposal. Furthermore, few patients voiced concerns on the environmental impact of single-use materials and medication waste like unused doses. Patients also discussed privacy concerns related to refrigeration and sharps like visitors or co-workers spotting sharps bins or medicines in the fridge. For some patients ‘*trying to get rid of the yellow [sharps] bin was like a symbol of like failure*’ (PAT9) after an unsuccessful cycle.

‘*Because a lot of people… are keen to not let people know for fear of it not working.* *So it's the last thing you want is a whole fridge in your kitchen full of like all the different IVF medications, and you have somebody around for tea.*’ (HCP1, pharmacist).

Participants imagined that MAPs would simplify logistics, reduce stress, and increase patients’ privacy if sharps and the need for refrigeration were removed. However, one patient highlighted that this would only apply if all injections were replaced. HCPs also welcomed sharps removal as it could simplify their disposal processes. Furthermore, few participants appreciated MAP’s potential waste reduction with no consumables being required but recommended to consider disposal strategies.

‘*I think it'd be more accessible for them [MAPs] to leave the house. You know, you wouldn't worry as much about carrying that little fridge pack that I carry out.*’ (PAT2, patient).

Finally, multiple patients emphasized their gratitude for financial support from public schemes and highlighted that MAPs should come at a similar cost to injections.

### Theme 5: Education and training - healthcare professionals and patients

Participants discussed education from the perspective of patient training and HCP’s knowledge. Patients highlighted the value of comprehensive injection training considering their emotional and financial investment. Demonstration sessions were provided by nurses in the clinic, community pharmacists, or both. Many patients appreciated the opportunity to practise with demonstration devices, step-by-step guides and videos. Some patients suggested improvements like more information on injection site rotation and potential errors.

Patients reported that counselling by HCPs reduced anxiety, however, poor knowledge might instill distrust in the profession. Some patients voiced frustration with *‘a big variety in… advice and information*’ (PAT5) by community pharmacists. On the other side, pharmacists highlighted little information on prescriptions, complex treatment regimens, unlicensed use and sparse professional education as barriers to successful counselling. Some pharmacists also expressed interest in more interprofessional cooperation with fertility clinics.

‘*It would be good for the pharmacies to have the regime that the patients are working off rather than us relying on the patient… it doesn't instil confidence I think if we're questioning certain things.*’ (HCP1, pharmacist).

HCPs imagined that counselling patients on the use of MAPs would be easier than injection training and save time. Nevertheless, patient education was considered essential for acceptance of MAPs and should include measures to address potential errors like MAPs coming off the administration site.

‘*The counselling would be shorter and less cumbersome.*’ (HCP2, pharmacist).

## Discussion

This study explored patients’, partners’ and HCP’s experiences of fertility injections and their perspectives on the potential use of MAPs. The handling and administration of injections posed some practical and emotional challenges for patients. However, the wish for treatment success increased patients’ resilience and willingness to endure difficulties. Participants were generally interested in MAPs and anticipated an improved patient experience compared to injectables, particularly for long-term use. Comparable cost, equal efficiency, comprehensive counselling and good handling properties were determined as important enablers to potential MAP use in fertility care.

Consistent with prior work on fertility treatments, patients were impacted by injections both physically and emotionally. They faced challenges in preparing and administering injections including reconstitution [8–10], bruises [36], fear of mistakes [10], and anxiety to self-inject. [6] These experiences are not unique to fertility care but have also been reported for self-injection in other therapeutic areas like diabetes [37, 38], psoriasis [39], inflammatory bowel disease and multiple sclerosis. [40, 41] Furthermore, identified drivers for MAP acceptance like ease of use, discretion, and pain reduction aligned with previous research on MAP-based vaccination [21, 23, 24], contraception [28, 31] and HIV prevention [30]. Interviewees also shared concerns around skin irritation [21, 27, 42, 43] and safety of MAP materials. [31].

Many participants emphasized treatment success as being critical to decision-making in fertility care. This observation aligned with previous discrete choice experiments that reported efficacy, risk of complications and cost to significantly impact patients’ treatment choice. [44–48] Efficiency was also crucial to the acceptance of MAP-based vaccination. [21, 23] While patients tend to approach new medicines with caution as reported for novel treatments like gene therapy [49, 50] and switches from originator biologics to biosimilars [51], the aversion towards MAPs may be particularly prominent among fertility patients due to their fear of treatment failure.

Most participants in past studies preferred MAPs over injections [17, 22, 24, 25, 31], while some favored the injection due to its familiarity or local skin reactions from MAPs. [17, 24, 25] However, these studies compared MAPs to HCP-delivered injections whereas in fertility care patients usually self-administer. In our study, patients discussed their choice depending on personality traits, prior experiences and cost if MAPs were equally effective. This aligned with former work highlighting comparable pricing of contraceptive MAPs to be essential for their acceptance. [31] However, decisions in fertility care may be more complex and could depend on the treatment regimen as some patients were only interested in MAPs if replacing all injections.

Furthermore, discussed logistical and lifestyle adjustments like changes to social schedules, medication storage, and sharps disposal corresponded with prior work on injections in fertility care [7–9] and other therapeutic areas like osteoporosis [52] and diabetes. [53, 54] Positive effects of room-temperature storage were previously described for MAP-based vaccination but largely focussed on logistical advantages regarding medicine distribution. [23] As highlighted in our study, removing refrigeration may have a logistical and emotional impact on fertility patients as it enhances discretion and flexibility. Further, participants critically appraised the environmental impact of medication waste which may reflect an increasing interest in sustainable healthcare solutions. [55–57] While policymakers in Kenya previously raised environmental concerns of improper MAP disposal [30], participants in our study anticipated a positive impact through the reduction of single-use materials.

Corresponding with former findings that reported better training to reduce injection burden and treatment interference with daily life [7, 58], patient support and training were identified as key facilitators to successful treatment. Also, psychological support helped to manage complex protocols and treatment failure previously. [59, 60] Clinical guidelines for fertility management described psychosocial support as critical [61] and recommend that counselling should be offered before, during and after fertility treatment irrespective of its outcomes. [62] Moreover, participants in former MAP acceptability studies highlighted the importance of comprehensive training and clear instructions to ensure correct use of MAPs. [16, 23, 28] While education is critical to successfully implement any new medicine, patients’ vulnerability and fear of treatment failure may pose unique support requirements for MAPs in fertility care.

Moreover, different aspects of involving partners in fertility injections were discussed previously including shared intimacy [36] and increased injection burden. [7] While MAPs may reduce patients’ dependency on others for help, partners could participate in their administration as highlighted in our study. Oppositely, women were reluctant to involve their partners in applying contraceptive MAPs as they questioned their ability to administer them correctly. [28] While involving partners in fertility treatments is important [63], their wish to participate in MAP administration may be unique to fertility care.

To our knowledge, this is the first study to explore patients’ and HCP’s perspectives on potential MAP-based fertility treatments. While largely aligning with previous research on other MAP applications, some unique requirements of fertility patients were noted resulting from complex treatment regimens and high hopes for a positive outcome. Hence, time and comprehensive education may be required to overcome patients’ apprehension to try MAPs. Moreover, the wish of partners to get involved in drug administration should be acknowledged.

Additionally, this study has implications for the development of future injectables. Many participants emphasized the importance of patient perspectives when designing new medicines. Particularly, ready-to-use dosage forms, short-term room temperature storage, and sensible pack sizes that streamline handling and minimize waste were discussed.

### Strengths and limitations

The strengths of this study lay in gathering diverse perspectives including both patient and healthcare professional participants and in working in an interprofessional research team between clinical pharmacy and pharmaceutics. This enabled researchers to discuss study design and findings from different perspectives, helping to identify assumptions and biases throughout the study and enhancing the study’s rigor.

Some limitations of this study should be noted. Sample size was small with 11 participants interviewed per group (patients/partners, healthcare professionals). However, efforts were made to recruit participants of varying demographics and experience with fertility treatment to ensure that diverse perspectives were included and a representative sample obtained. All participants were based in Ireland which may limit the transferability of the findings to other locations with different healthcare systems. All patient participants had a university degree, only one interviewee had a medical card, and most HCPs were female and practiced in an urban/suburban location. While some of these traits like a highly educated patient and predominately female HCP cohort, are reflective of the respective population, the study may not have captured the full diversity of patient and HCP perspectives. Furthermore, study results might be influenced by a self-selection bias as patients and HCPs that are open to new medicines may have been more inclined to participate. Although the concept of MAPs was explained in detail and placebo samples shown, acceptability studies that allow participants to test placebo MAPs may provide additional insights for their use in fertility care.

## Conclusion

This study explored patients’, partners’ and HCP’s experiences of injections and their perspectives on potential MAP use in fertility care. Most patients became accustomed to the handling and administration of injections quickly. Nevertheless, they posed some practical and emotional challenges including reconstitution, sharps disposal, fear of mistakes, and management of anticipated outcomes. Overall, participants were interested in MAPs and positively highlighted their potential straightforward use, minimal preparation requirements and little administration pain. Some participants noted that MAPs may be primarily relevant to long-term injections and specific patient groups. Furthermore, an adjustment period could be required as many fertility patients were apprehensive to trying new medicines. Therefore, comprehensive counselling was considered critical to educate patients on MAPs. While the acceptability of MAPs was investigated for other indications, this study highlighted their potential positive impact on the unique practical and emotional challenges of fertility patients. The findings will help to inform next steps in exploring MAP-based fertility treatments.

## Supplementary Information

Below is the link to the electronic supplementary material.

Supplementary file1 (DOCX 57 kb)

## Data Availability

Access to the generated datasets is limited to the authors of this study as approved by the Social Research Ethics Committee of University College Cork.

## References

[1] Vander Borght M, Wyns C. Fertility and infertility: definition and epidemiology. Clin Biochem. 2018;62:2–10. 10.1016/j.clinbiochem.2018.03.012. 29555319

[2] The European IVF Monitoring Consortium (EIM) for the European Society of Human Reproduction and Embryology (ESHRE), Smeenk J, Wyns C, De Geyter C, et al. ART in Europe, 2019: results generated from European registries by ESHRE†. Hum Reprod. 2019;38(12):2321–38. 10.1093/humrep/dead197. PMC10694409 37847771

[3] Adamson GD, Creighton P, Mouzon J, et al. How many infants have been born with the help of assisted reproductive technology? Fertil Steril. 2025;124(1):40–50. 10.1016/j.fertnstert.2025.02.009. 39947276

[4] Collura B, Hayward B, Modrzejewski KA, et al. Identifying factors associated with discontinuation of infertility treatment prior to achieving pregnancy: results of a nationwide survey. J Patient Exp. 2024;1(11):23743735241229380. 10.1177/23743735241229380. PMC10898306 38414755

[5] Boivin J, Takefnan JE. Impact of the in-vitro fertilization process on emotional, physical and relational variables*. Hum Reprod. 1996;11(4):903–7. 10.1093/oxfordjournals.humrep.a019276. 8671350

[6] Barrière P, Avril C, Benmahmoud-Zoubir A, et al. Patient perceptions and understanding of treatment instructions for ovarian stimulation during infertility treatment. Reprod Biomed Soc Online. 2019;9:37–47. 10.1016/j.rbms.2019.08.003. PMC6976931 31993512

[7] Brod M, Fennema H. Validation of the controlled ovarian stimulation impact measure (COSI): assessing the patient perspective. Health Qual Life Outcomes. 2013;11:130. 10.1186/1477-7525-11-130. PMC3734046 23902854

[8] Brod M, Verhaak CM, Wiebinga CJ, et al. Improving clinical understanding of the effect of ovarian stimulation on women’s lives. Reprod Biomed Online. 2009;18(3):391–400. 10.1016/s1472-6483(10)60098-3. 19298739

[9] Lankreijer K, D’Hooghe T, Sermeus W, et al. Development and validation of the FertiMed questionnaire assessing patients’ experiences with hormonal fertility medication. Hum Reprod. 2016;31(8):1799–808. 10.1093/humrep/dew111. 27220980

[10] Huisman D, Raymakers X, Hoomans E. Understanding the burden of ovarian stimulation: fertility expert and patient perceptions. Reprod Biomed Online. 2009;1(19):5–10. 10.1016/S1472-6483(10)60271-4. 19891842

[11] Welcker JT, Nawroth F, Bilger W. Patient evaluation of the use of follitropin alfa in a prefilled ready-to-use injection pen in assisted reproductive technology: an observational study. Reprod Biol Endocrinol. 2010;8:111. 10.1186/1477-7827-8-111. PMC2949697 20843323

[12] Landfeldt E, Jablonowska B, Norlander E, et al. Patient preferences for characteristics differentiating ovarian stimulation treatments. Hum Reprod. 2012;27(3):760–9. 10.1093/humrep/der449. 22240210

[13] Leão RdeBF, Esteves SC. Gonadotropin therapy in assisted reproduction: an evolutionary perspective from biologics to biotech. Clinics (Sao Paulo). 2014;69(4):279–93. 10.6061/clinics/2014(04)10. PMC3971356 24714837

[14] Liu M, Svirskis D, Proft T, et al. Progress in peptide and protein therapeutics: challenges and strategies. Acta Pharm Sin B. 2025;15(12):6342–81. 10.1016/j.apsb.2025.10.026. PMC12750180 41477339

[15] Bajracharya R, Song JG, Back SY, et al. Recent advancements in non-invasive formulations for protein drug delivery. Comput Struct Biotechnol J. 2019;17:1290–308. 10.1016/j.csbj.2019.09.004. PMC6944732 31921395

[16] Birchall JC, Clemo R, Anstey A, et al. Microneedles in clinical practice–an exploratory study into the opinions of healthcare professionals and the public. Pharm Res. 2011;28(1):95–106. 10.1007/s11095-010-0101-2. 20238152

[17] Arya J, Henry S, Kalluri H, et al. Tolerability, usability and acceptability of dissolving microneedle patch administration in human subjects. Biomaterials. 2017;128:1–7. 10.1016/j.biomaterials.2017.02.040. PMC5382793 28285193

[18] Forster AH, Witham K, Depelsenaire ACI, et al. Safety, tolerability, and immunogenicity of influenza vaccination with a high-density microarray patch: results from a randomized, controlled phase I clinical trial. PLoS Med. 2020;17(3):e1003024. 10.1371/journal.pmed.1003024. PMC7077342 32181756

[19] Sanjanwala D, Shinde A, Patravale V. Formulation, sterilization, and clinical evaluation of microneedles for vaccine and biologic delivery: a review. Int J Pharm. 2025;682:125874. 10.1016/j.ijpharm.2025.125874. 40544976

[20] Gopal K, Khalid R, Mahmood S. Dissolvable microneedles with their design, materials, and limitations in translation: a technical review. AAPS PharmSciTech. 2025;27(1):13. 10.1208/s12249-025-03246-w. 41184649

[21] Marshall S, Fleming A, Moore AC, et al. Acceptability of microneedle-patch vaccines: a qualitative analysis of the opinions of parents. Vaccine. 2017;35(37):4896–904. 10.1016/j.vaccine.2017.07.083. 28780122

[22] Frew PM, Paine MB, Rouphael N, et al. Acceptability of an inactivated influenza vaccine delivered by microneedle patch: results from a phase I clinical trial of safety, reactogenicity, and immunogenicity. Vaccine. 2020;38(45):7175–81. 10.1016/j.vaccine.2020.07.064. 32792250

[23] Jacoby E, Jarrahian C, Hull HF, et al. Opportunities and challenges in delivering influenza vaccine by microneedle patch. Vaccine. 2015;33(37):4699–704. 10.1016/j.vaccine.2015.03.062. 25842218

[24] Griffin P, Elliott S, Krauer K, et al. Safety, acceptability and tolerability of uncoated and excipient-coated high density silicon micro-projection array patches in human subjects. Vaccine. 2017 4;35(48, Part B):6676–84. 10.1016/j.vaccine.2017.10.021 29074201

[25] Fernando GJP, Hickling J, Jayashi Flores CM, et al. Safety, tolerability, acceptability and immunogenicity of an influenza vaccine delivered to human skin by a novel high-density microprojection array patch (Nanopatch™). Vaccine. 2018;36(26):3779–88. 10.1016/j.vaccine.2018.05.053. 29779922

[26] Mooney K, McElnay JC, Donnelly RF. Parents’ perceptions of microneedle-mediated monitoring as an alternative to blood sampling in the monitoring of their infants. Int J Pharm Pract. 2015;23(6):429–38. 10.1111/ijpp.12195. 25807981

[27] Mooney K, McElnay JC, Donnelly RF. Paediatricians’ opinions of microneedle-mediated monitoring: a key stage in the translation of microneedle technology from laboratory into clinical practice. Drug Deliv Transl Res. 2015;5(4):346–59. 10.1007/s13346-015-0223-5. 25787733

[28] El-Sahn M, Elliott R, El-Sahn M, et al. End-user research into understanding perceptions of and reactions to a microarray patch (MAP) for contraception among women in Ghana, Kenya and Uganda. Front Reprod Health. 2024;7(6):1351692. 10.3389/frph.2024.1351692PubMedPMID:38515793;PubMedCentralPMCID:PMC10954799. PMC10954799 38515793

[29] Quinn HL, Hughes CM, Donnelly RF. In vivo and qualitative studies investigating the translational potential of microneedles for use in the older population. Drug Deliv Transl Res. 2018;8(2):307–16. 10.1007/s13346-017-0393-4. PMC5830461 28508376

[30] Gachigua SG, Karuga R, Ngunjiri A, et al. Microarray patch for HIV prevention and as a multipurpose prevention technology to prevent HIV and unplanned pregnancy: an assessment of potential acceptability, usability, and programmatic fit in Kenya. Front Reprod Health. 2023;5:1125159. 10.3389/frph.2023.1125159. PMC10164997 37168102

[31] Brunie A, Callahan RL, Godwin CL. User preferences for a contraceptive microarray patch in India and Nigeria: qualitative research on what women want. PLoS ONE. 2019;14(6):e0216797. 10.1371/journal.pone.0216797. PMC6553846 31170173

[32] Tong A, Sainsbury P, Craig J. Consolidated criteria for reporting qualitative research (COREQ): a 32-item checklist for interviews and focus groups. Int J Qual Health Care. 2007;19(6):349–57. 10.1093/intqhc/mzm042. 17872937

[33] Hennink M, Kaiser BN. Sample sizes for saturation in qualitative research: a systematic review of empirical tests. Soc Sci Med. 2022;292:114523. 10.1016/j.socscimed.2021.114523. 34785096

[34] Braun V, Clarke V. Using thematic analysis in psychology. Qual Res Psychol. 2006;3(2):77–101. 10.1191/1478088706qp063oa.

[35] Lumivero. 2026; NVivo (Version 15.5.2) [Computer software, RRID SCR_014802]. https://lumivero.com/products/nvivo/

[36] Silva S, Machado H. Heterosexual couples’ uses and meanings of ovarian stimulation: relatedness, embodiment and emotions. Health (N Y). 2011;15(6):620–32. 10.1177/1363459310364161. 21177709

[37] Ellis K, Mulnier H, Forbes A. Perceptions of insulin use in type 2 diabetes in primary care: a thematic synthesis. BMC Fam Pract. 2018;19(1):70. 10.1186/s12875-018-0753-2. PMC5964885 29788908

[38] Abu Hassan H, Tohid H, Mohd Amin R, et al. Factors influencing insulin acceptance among type 2 diabetes mellitus patients in a primary care clinic: a qualitative exploration. BMC Fam Pract. 2013;14(1):164. 10.1186/1471-2296-14-164. PMC4231611 24164794

[39] Zaino M, Parks E, McNeil J, et al. Patients’ preferences regarding modes of systemic psoriasis treatment – a qualitative study. J Psoriasis Psoriatic Arthritis. 2024;9(4):136–42. 10.1177/24755303241253200. PMC11457890 39381162

[40] Puzovic M, Morrissey H, Ball PA. The Experience of Home Parenteral Therapy: A Thematic Analysis of Patient Interviews. Pharmacy. 2023;11(5):133. 10.3390/pharmacy11050133. PMC10514802 37736903

[41] Capelle A de C van, Meide H van der, Vosman FJH et al., A qualitative study assessing patient perspectives in the process of decision-making on disease modifying therapies (DMT’s) in multiple sclerosis (MS). PLOS ONE. 2017 12(8): e0182806. 10.1371/journal.pone.0182806 PMC5570319 28837609

[42] Mooney K, McElnay JC, Donnelly RF. Children&s views on microneedle use as an alternative to blood sampling for patient monitoring. Int J Pharm Pract. 2014;22(5):335–44. 10.1111/ijpp.12081. 24308565

[43] Caffarel-Salvador E, Tuan-Mahmood TM, McElnay JC, et al. Potential of hydrogel-forming and dissolving microneedles for use in paediatric populations. Int J Pharm. 2015;489(1):158–69. 10.1016/j.ijpharm.2015.04.076. 25940042

[44] Musters AM, de Bekker-Grob EW, Mochtar MH, et al. Women’s perspectives regarding subcutaneous injections, costs and live birth rates in IVF. Hum Reprod. 2011;26(9):2425–31. 10.1093/humrep/der177. 21672926

[45] Palumbo A, De La Fuente P, Rodríguez M, et al. Willingness to pay and conjoint analysis to determine women’s preferences for ovarian stimulating hormones in the treatment of infertility in Spain. Hum Reprod. 2011;26(7):1790–8. 10.1093/humrep/der139. PMC3113505 21558333

[46] van den Wijngaard L, Rodijk ICM, van der Veen F, et al. Patient preference for a long-acting recombinant FSH product in ovarian hyperstimulation in IVF: a discrete choice experiment. Hum Reprod. 2015;30(2):331–7. 10.1093/humrep/deu307. 25432926

[47] Von Estorff F, Mochtar MH, Van Lehmann V, et al. Driving factors in treatment decision-making of patients seeking medical assistance for infertility: a systematic review. Hum Reprod Update. 2024;30(3):341–54. 10.1093/humupd/dmae001. PMC11063545 38305635

[48] Skedgel C, Ralphs E, Finn E, et al. How do people with experience of infertility value different aspects of assistive reproductive therapy? Results from a multi-country discrete choice experiment. Patient Patient-Centered Outcomes Res. 2022;15(4):459–72. 10.1007/s40271-021-00563-7. PMC9197909 34940935

[49] Bhatt N, Anderson T, Bryndziar T, et al. Perceptions and recommendations regarding haemophilia B gene therapy: a multistakeholder view from patients, caregivers and healthcare professionals. Haemophilia. 2026;32(4):960–73. 10.1111/hae.70314. PMC13378621 42117390

[50] Pietu G, Giraud N, Chamouard V, et al. Perspectives and perception of haemophilia gene therapy by French patients. Haemophilia. 2024;30(1):68–74. 10.1111/hae.14908. 38058235

[51] Varma M, Almarsdóttir AB, Druedahl LC. “Biosimilar, so it looks alike, but what does it mean?” A qualitative study of Danish patients’ perceptions of biosimilars. Basic Clin Pharmacol Toxicol. 2022;130(5):581–91. 10.1111/bcpt.13719. PMC9314148 35261174

[52] Press D. Understanding compliance issues for daily self-injectable treatment in ambulatory care settings. Patient Prefer Adherence. 2008;6(2):129–36. PMC2770412 19920953

[53] Ndara FN, Nuuyoma V. Patients’ lived experiences of self-injectable diabetes treatment: a phenomenological study. Health SA Gesondheid. 2023;28:2359. 10.4102/hsag.v28i0.2359. PMC10659562 38021208

[54] Liang W, Lo SHS, Chow KM, et al. Perception of self-management and glycaemic control in people with type 2 diabetes receiving insulin injection therapy: a qualitative study. Prim Care Diabetes. 2023;17(6):587–94. 10.1016/j.pcd.2023.08.006. 37658019

[55] Scholz F, Börner N, Schust SA, et al. Focus on patient perspectives in climate action policies for healthcare. A German survey analysis on what patients are willing to do. Front Public Health. 2024;12:1477313. 10.3389/fpubh.2024.1477313. PMC11629199 39659718

[56] Legrand J, Aubin-Auger I, De Bary L, et al. Sustainable development in general practice. Fam Pract. 2023;40(3):511–8. 10.1093/fampra/cmad003. 36652286

[57] Beckwith SE, Petit HJ, Sullivan EE, et al. Patient perceptions about the environmental impact of hospitals and surgical care. Am Surg. 2025;91(12):2090–5. 10.1177/00031348251346528. 40437686

[58] Tahta T, Kaya Y, Tokgöz VY, et al. The effect of infertility support education on treatment outcomes in women with unexplained infertility. J Comp Eff Res. 2026;15(3):e250159. 10.57264/cer-2025-0159PubMedPMID:41705997;PubMedCentralPMCID:PMC12976645. PMC12976645 41705997

[59] Wyns C, Blockeel C, Guivarc’h-Lévêque A, et al. Current practices and challenges in assisted reproductive technology care pathways in France and Belgium: the AMPLITUDE survey. Front Reprod Health. 2025 30;7:1617628. 10.3389/frph.2025.1617628 PMC12518220 41098623

[60] Domar AD, Rooney K, Hacker MR, Sakkas D, Dodge LE. Burden of care is the primary reason why insured women terminate in vitro fertilization treatment. Fertil Steril. 2018;109(6):1121–6. 10.1016/j.fertnstert.2018.02.130. PMC6080203 29935647

[61] World Health Organization. Guideline for the prevention, diagnosis and treatment of infertility [Internet]. Geneva; 2025 [cited 2026 Jul 27]. Available from: https://www.who.int/publications/i/item/9789240115774 41587292

[62] Petch S, O’Byrne L, Hartigan L, et al. National Clinical Practice: Guideline In Vitro Fertilisation (IVF) and Intracytoplasmic Sperm Injection (ICSI). Nat Women Infants Health Progr Inst Obstet Gynaecol. 2025;19(3):237–48.

[63] Sylvest R, Schmidt L, Pinborg A, et al. Emotional experience of Danish men during fertility treatment: qualitative study on engagement, hope and loss. Reprod Biomed Online. 2026;52(2):105217. 10.1016/j.rbmo.2025.105217. 41448982

