# Supplementary material for: Microarray patches as a potential alternative to injectable fertility medications: a qualitative study of patients’ and healthcare professionals’ perceptions
Source: Int J Clin Pharm. 2026 Sep 23;48(6):2429–39. doi: 10.1007/s11096-026-02220-1 (PMC13731474; doi:10.1007/s11096-026-02220-1)
Supplement: Supplementary file 1 [file 11096_2026_2220_MOESM1_ESM.docx]

# Supplemental material

## Pre-approved topic guides

### Patients

*Introduction: Thank you for taking the time to talk to me today. I am interested in hearing about your experience with IVF injections. I also want to introduce a new way of taking medicines to you and hear your thoughts on that. The interview will be audio-recorded to make sure all answers are accurately documented. There are no right or wrong answers to the questions, and no one will know your specific answers to them. So please feel free to be as open and honest as you feel comfortable.*

*Part 1: Current use of subcutaneous IVF injections*

*In part one, I would like to learn about your experiences with injecting IVF medicines.*

1. Were you prescribed IVF medicines for injection previously?
2. What type of injection was it? I have some examples here. (Show patient prefilled syringe, pen, and vial for reconstitution with syringe to help identify relevant injection types.)
3. Were you injecting the IVF medicines yourself?
(a) If self-injecting:

- How did you approach self-injecting? (prompt- How did you prepare? What steps did you have in place?)
- Did you feel comfortable during the injection? If not, what were the reasons that made you feel uncomfortable?
- Is there anything that you do not like about the IVF injections?
- Were there differences regarding your experience in using different types of injections (prefilled syringe, pen)?

(b) If not self-injecting:

- Why were you not injecting the medicines yourself?
- Who was injecting the medicines for you?
- Can you describe the routine you had for injecting?
- Were there differences regarding your experience in using different types of injections (prefilled syringe, pen)?

4. Did the injections affect your daily routine?
5. Any other observations about the experience of injecting that you feel would be worth highlighting?

*Part 2: Microneedles as a non-invasive form of self-administration*

*Let’s move on to part two. Here, I want to introduce a new way of taking medicines to you and hear your thoughts on it. While it is long known that injections can be very challenging for patients, it is difficult to take IVF medications in a different way. They are very sensitive products so they may be destroyed in the stomach, hence we cannot put them in a tablet. Because of their special properties, they cannot be applied as a cream or patch either because not enough of the medicine can go through the natural barrier of the skin.*

*However, one option to overcome this problem are microneedles. Here I have an example for you of what that is without any drugs (Show patient a placebo microneedle patch). They are very tiny needles, so you can barely see them attached to a patch. They are called needles as they help the medicine to overcome the natural barrier of the skin but because they are so tiny you cannot feel them. Another advantage of microneedles compared to most IVF injections is that they may be stored at room temperature rather than in the fridge.*

6. Do you understand the concept of microneedles? Do you have any questions on the microneedle concept?
7. Do you think you would be comfortable applying a microneedle patch? How do you imagine that would compare to current IVF injections? And why?
8. If a microneedle patch was available for IVF instead of injections, would you prefer using the patch? Which of the following differences between microneedle patches and injections would be relevant to you?

- Storage at room temperature compared to storage in the fridge
- No separate disposal of sharps in a sharps bin required
- Smaller size
- Convenience of application including discretion
- Reduced training needs
- Confidence in self-administration

9. Have you any additional thoughts on the potential use of microneedles for IVF that you would like to share with us today?

*Conclusion: That brings us to the end of the interview today. How did you find the experience? How are you feeling?*

*Thank you very much again for taking the time to talk to me today. If you feel distressed at any stage after the interview, you have our contact details to reach out to the research team. You can also contact your GP or visit the HSE website for mental support by using the information provided in the information sheets.*

### Partners

*Introduction: Thank you for taking the time to talk to me today. I am interested in hearing about your experience with IVF injections. I also want to introduce a new way of taking medicines to you and hear your thoughts on that. The interview will be audio-recorded to make sure all answers are accurately documented. There are no right or wrong answers to the questions, and no one will know your specific answers to them. So please feel free to be as open and honest as you feel comfortable.*

*Part 1: Current use of subcutaneous IVF injections*

*In part one, I would like to learn about your and your partner’s experiences with injecting IVF medicines.*

1. Was your partner prescribed IVF medicines for injection previously?
2. What type of injection was it? I have some examples here. (Show prefilled syringe, pen, and vial for reconstitution with syringe to help identify relevant injection types.)
3. Have you injected IVF medicines for your partner before?
(a) If injecting IVF medicines for your partner:
    - Why is/was your partner not injecting the medicines themselves?
    - Can you describe the routine you had for injecting?
    - Did you find the injection of IVF medicines difficult or burdensome? If so, why did you find the injections difficult?
    - Were there differences regarding your experience in using different types of injections (prefilled syringe, pen)?
(b) If your partner is self-injecting their IVF medicines:
    - Do you think they struggled with these injections sometimes? If so, what were the main reasons for their struggle?

- Were there differences regarding your experience in using different types of injections (prefilled syringe, pen)?

4. Did the injections affect your partner’s daily routine? Or your daily routine?
5. Any other observations about the experience of injecting that you feel would be worth highlighting?

*Part 2: Microneedles as a non-invasive form of self-administration*

*Let’s move on to part two. Here, I want to introduce a new way of taking medicines to you and hear your thoughts on it. While it is long known that injections can be very challenging for patients, it is difficult to take IVF medications in a different way. They are very sensitive products so they may be destroyed in the stomach, hence we cannot put them in a tablet. Because of their special properties, they cannot be applied as a cream or patch either because not enough of the medicine can go through the natural barrier of the skin.*

*However, one option to overcome this problem are microneedles. Here I have an example for you of what that is without any drugs (Show partner a placebo microneedle patch). They are very tiny needles, so you can barely see them attached to a patch. They are called needles as they help the medicine to overcome the natural barrier of the skin but because they are so tiny you cannot feel them. Another advantage of microneedles compared to most IVF injections is that they may be stored at room temperature rather than in the fridge.*

6. Do you understand the concept of microneedles? Do you have any questions on the microneedle concept?
7. Do you think you and your partner would be comfortable applying a microneedle patch? How do you imagine that would compare to current IVF injections? And why?
8. If a microneedle patch was available for IVF instead of injections, do you think you and your partner would prefer using the patch? Which of the following differences between microneedle patches and injections would be relevant to you and your partner?

- Storage at room temperature compared to storage in the fridge
- No separate disposal of sharps in a sharps bin required
- Smaller size
- Convenience of application including discretion
- Reduced training needs
- Confidence in self-administration

9. Have you any additional thoughts on the potential use of microneedles for IVF that you would like to share with us today?

*Conclusion: That brings us to the end of the interview today. How did you find the experience? How are you feeling?*

*Thank you very much again for taking the time to talk to me today. If you feel distressed at any stage after the interview, you have our contact details to reach out to the research team. You can also contact your GP or visit the HSE website for mental support by using the information provided in the information sheets.*

### Healthcare professionals

*Introduction: Thank you for taking the time to talk to me today. I am interested in hearing about your experiences with patients self-injecting IVF medicines. I also want to introduce a new way of taking medicines to you and hear your thoughts on that. The interview will be audio-recorded to make sure all answers are accurately documented. There are no right or wrong answers to the questions, and no one will know your specific answers to them. So please feel free to be as open and honest as you feel comfortable.*

*Part 1: Subcutaneous injections in IVF treatment*

*In part one, I would like to learn about your experiences with patients that inject IVF medicines.*

1. Has a patient ever complained to you about the injection of IVF medicines? And for what reasons?
2. Are there certain product types (prefilled syringe, vial for reconstitution, pen) that patients particularly struggle with?
3. How do you usually counsel the patient when they are starting with IVF injections e.g. how do you train the patients?
4. Has a patient ever talked to you about stopping IVF treatment because of the injections?
5. Has a patient ever told you about IVF injections affecting their daily routine?
6. Any other observations about the experience of injecting that you feel would be worth highlighting?

*Part 2: Microneedles as a non-invasive form of self-administration*

*Introduction: Let’s move on to part two. Here, I want to introduce a new way of taking medicines to you and hear your thoughts on it. While it is long known that injections can be very challenging for patients, it is difficult to take IVF medications in a different way. They are very sensitive products so they may be destroyed in the stomach, hence we cannot put them in a tablet. Because of their special properties, they cannot be applied as a cream or patch either because not enough of the medicine can go through the natural barrier of the skin.*

*However, one option to overcome this problem are microneedles. Here I have an example for you of what that is without any drugs (Show participant a placebo microneedle patch). They are very tiny needles, so you can barely see them attached to a patch. They are called needles as they help the medicine to overcome the natural barrier of the skin but because they are so tiny a patient cannot feel them. Another advantage of microneedles compared to most IVF injections is that they may be stored at room temperature rather than in the fridge.*

7. Do you understand the concept of microneedles? Do you have any questions on the microneedle concept?
8. Do you think patients would feel comfortable applying a microneedle patch for IVF treatment? How do you imagine that would compare to current IVF injections? And why?
9. If a microneedle patch was available for IVF instead of injections, do you think patients would prefer using the patch? Which of the following differences between microneedle patches and injections would be most relevant in your opinion?

- Storage at room temperature compared to storage in the fridge
- No separate disposal of sharps in a sharps bin required
- Smaller size
- Convenience of application including discretion
- Reduced training needs
- Confidence in self-administration

10. Have you any additional thoughts on the potential use of microneedles for IVF that you would like to share with us today?

*Conclusion: That brings us to the end of the interview today. How did you find the experience? How are you feeling?*

*Thank you very much again for taking the time to talk to me today. If you feel distressed at any stage after the interview, you have our contact details to reach out to the research team. You can also contact your GP or visit the HSE website for mental support by using the information provided in the information sheets.*

## Table S1 Additional selection of quotes

**Theme 1: The complexity of administration - drug and formulation considerations**

‘*When I got the pen needle, I was thinking this is great and… when I had to go back to the traditional thing of attaching the needle… I was thinking why hasn’t this company just come up with a pen?*’ (PAT10, patient)

‘*Especially when I was pregnant and on injections for 14 weeks, I found that my stomach I was running out of places to inject.*’ (PAT5, patient)

‘*It scares them a lot that if they get it wrong, they'll cause themselves harm, but also that if they get it wrong, it won't be a successful cycle.*’ (HCP11, pharmacist)

‘*Once the pen malfunctioned on me and I say god, do I give myself a second dose? What happened? Will I end up with hyperstimulation if I give myself another dose?*’ (PAT3, patient)

‘*I can't really see any patient favouring an injection over a microneedle patch, with the exception of maybe allergy to the adhesive or site reaction that physically means they can't use the microneedle patch.*’ (HCP2, pharmacist)

‘*What are they [MAPs] made of? Especially if they're going to be dissolved in the skin. Like is it harmful? Is it dangerous?*’ (HCP10, midwife)

**Theme 2: The importance of support systems - partners, peers, and healthcare professionals**

‘*I would definitely say as a solo person, it's definitely much harder because you can't guarantee you have someone to help you.’* (PAT2, patient)

‘*I had an amazing pharmacist… Only for her I wouldn't have been able to do any of this.*’ (PAT11, patient)

‘*You just become a bit of a carer for your partner. It's not a very pleasant situation to be honest with you.*’ (PAT1, patient partner)

‘*He [partner] didn't like seeing me do it. So that was a little bit isolating.*’ (PAT6, patient)

‘*It's a psychological aspect of it. It's very, very draining on the relationship… we went through two rounds of IVF. I can't imagine we would have been able to go through three or four.*’ (PAT1, patient’s partner)

‘Y*ou don't want to end up in a situation where… somebody arrived home half an hour late to no fault of their own and all of a sudden there is, you know, contention in the couple because of it. So, I always advise people do the injections yourself.*’ (HCP5, nurse)

**Theme 3: Managing expectations - medicine information and treatment outcome**

*‘You do what you have to do, and if it works, brilliant. But and if it doesn't, at least you can say I've done everything in my power to try.*’ (PAT9, patient)

‘*If it didn't work, you're going to look back over all the things that where you may be deviated and even if they make no difference, I suppose you want to make sure that you do everything right.*’ (PAT10, patient)

‘*If they [MAPs] had moved positioning, I think it would maybe increase their anxiety…* *Some patients I think would love these, but I do think the anxious patients might find these worse.*’ (HCP6, nurse)

*‘You're paying financially, but you're also paying for the cost of hope and the cost of your heart. And if you're going to be given medication… that's not equally efficient or inferior it wouldn't be ethical for a doctor to prescribe it just because they think that you could be in discomfort.*’ (PAT9, patient)

‘*Just from, I suppose, a medical point of view we'd worry a little bit about the actual absorption of the medication, how well it [MAP] might work.*’ (HCP3, nurse)

‘*I would find because I am a larger person… I would have to have a deeper subcut needle. So… I would worry with the microneedle that it wouldn't go deep enough for me to fully uptake the medications.*’ (PAT2, patient)

‘*But if on the whole your cycle has a different outcome even if the patch isn't the reason, you do attach some of the blame to the thing that has changed in that cycle.*’ (PAT5, patient)

‘*I would care more about that anecdotal kind of information… than reading a leaflet telling me XY and Z… I would be asking my clinic, can you tell me like did this [MAP] work for somebody else.’* (PAT5, patient)

**Theme 4: Making it work - logistical and lifestyle adjustments**

‘*It did affect our lives at the time. You know, we had to kind of just not do things in the evenings because we had to do this.*’ (PAT7, patient)

‘*It's a constant thought process in your head of like what time is it now, trying to get back to the house, making sure you do everything.*’ (PAT9, patient)

‘*You couldn't wear certain clothes like your stomach could be so tender and sore.*’ (PAT11, patient)

‘*I didn't live at home for the time I just used to bring them [injections] in a little refrigerated little pack thing that I bought online, and it was fine.*’ (PAT2, patient)

‘*I wouldn't have anticipated that at the start… but… as I was going through it, I was thinking that it's actually a real pain to have all of this waste material and no way to get rid of it.*’ (PAT10, patient)

‘*Sometimes the sharps bin if it's in a car, if it's in a work bag… people are kind of guessing… I think people do find that nosiness and curiosity challenging.*’ (HCP5, nurse)

‘*Say I've two injections usually, and now I'm going to have an injection and a patch. It's more or less the same for me if I have to do one injection or two, so I just do two, you know.*’ (PAT5, patient)

‘*If it was a choice between doing an injection that's free or having a patch that you pay for… I would actually pick the injection.*’ (PAT5, patient)

**Theme 5: Education and training - healthcare professionals and patients**

‘*Unless you're actually dealing with this on a daily basis, you're not actually going to know anything about it.*’ (HCP1, pharmacist)

‘*It can be a drastic thing for a woman who, say, is left without something that they really need… or it wasn't explained how to use it... And then it's taken wrong, and it completely ruins a full cycle, which leaves them another four to six weeks down the road before they can start treatment again*.’ (HCP8, pharmacist)

‘*A lot of that I've picked up from the same support groups or sitting and having to do the physical research myself because it's not something that, especially as pharmacists, that is really covered much in the education*.’ (HCP8, pharmacist)
